# Fetal-Derived MyD88 Signaling Contributes to Poor Pregnancy Outcomes During Gestational Malaria

**DOI:** 10.3389/fmicb.2019.00068

**Published:** 2019-01-29

**Authors:** Renato Barboza, Lutero Hasenkamp, André Barateiro, Oscar Murillo, Erika Paula Machado Peixoto, Flávia Afonso Lima, Aramys Silva Reis, Lígia Antunes Gonçalves, Sabrina Epiphanio, Claudio R. F. Marinho

**Affiliations:** ^1^Departamento de Ciências Biológicas, Universidade Federal de São Paulo, Diadema, Brazil; ^2^Departamento de Parasitologia, Instituto de Ciências Biomédicas, Universidade de São Paulo, São Paulo, Brazil; ^3^Departamento de Análises Clínicas e Toxicológicas, Faculdade de Ciências Farmacêuticas, Universidade de São Paulo, São Paulo, Brazil

**Keywords:** mouse model, pregnancy, placenta, malaria, MyD88, *Plasmodium berghei*

## Abstract

Placental malaria (PM) remains a severe public health problem in areas of high malaria transmission. Despite the efforts to prevent infection poor outcomes in *Plasmodium* endemic areas, there is still a considerable number of preterm births and newborns with low birth weight resulting from PM. Although local inflammation triggered in response to malaria is considered crucial in inducing placental damage, little is known about the differential influence of maternal and fetal immune responses to the disease progression. Therefore, using a PM mouse model, we sought to determine the contribution of maternal and fetal innate immune responses to PM development. For this, we conducted a series of cross-breeding experiments between mice that had differential expression of the MyD88 adaptor protein to obtain mother and correspondent fetuses with distinct genetic backgrounds. By evaluating fetal weight and placental vascular spaces, we have shown that the expression of MyD88 in fetal tissue has a significant impact on PM outcomes. Our results highlighted the existence of a distinct contribution of maternal and fetal immune responses to PM onset. Thus, contributing to the understanding of how inflammatory processes lead to the dysregulation of placental homeostasis ultimately impairing fetal development.

## Introduction

Placental malaria (PM) constitutes a serious public health issue in areas of high malaria transmission as pregnant women are particularly susceptible to *Plasmodium* infection (Fried and Duffy, 2017). Despite the significant efforts that have been done to prevent and treat malaria, every year a considerable number of newborns with low birth weight are still reported as a result of PM (Rogerson et al., 2018). In addition, malaria during pregnancy can affect the transplacental transfer of nutrients, which contributes to the impaired development of the fetus (Rogerson et al., 2007).

The negative consequences of PM are related to the accumulation of infected red blood cells (iRBCs) in the intervillous space of the placenta. During *P. falciparum* infections, this accumulation is mediated by the protein VAR2CSA (Doritchamou et al., 2012), a member of the *P. falciparum* erythrocyte membrane protein 1 (PfEMP1) that promotes the binding of iRBCs to the chondroitin sulfate A (CSA) (Fried and Duffy, 1996; Beeson et al., 2000). The adhesion of iRBCs to the placenta is sufficient to induce an inflammatory process mainly through the recruitment of inflammatory cells and the production of pro-inflammatory mediators. Also, the immune response can disrupt a normal angiogenic process that can progress and culminate in low birth weight and preterm birth (Brabin et al., 2004; Desai et al., 2007; Umbers et al., 2011).

The trigger of pro-inflammatory mediators could be induced by the activation of the innate immune system via Pattern Recognition Receptors (PRRs) that recognize the Pathogen-associated molecular pattern (PAMPs). One of the most relevant PRRs family are the Toll-like receptors (TLRs). These receptors act as immune sensors for microorganisms and internal danger signals (Kawai and Akira, 2010). It is known that *Plasmodium* components can activate the TLR pathway (Franklin et al., 2009). As an example, *Plasmodium* glycosylphosphatidylinositol (GPI) is recognized by TLR2 and TLR4 (Krishnegowda et al., 2005), cytosolic RNA by TLR7 (Baccarella et al., 2013; Wu et al., 2014), and hemozoin and DNA by TLR9 (Parroche et al., 2007; Pichyangkul et al., 2004; Coban et al., 2010). Additionally, several TLRs polymorphisms have been associated with increased susceptibility to PM (Mockenhaupt et al., 2006; Leoratti et al., 2008; Hamann et al., 2010). All TLRs, except the TLR3, use as adaptor molecule the Myeloid differentiation primary response 88 (MyD88) protein. Upon activation of a TLR, the MyD88 allows the recruitment of several other proteins, which lead to a signaling pathway downstream that culminate in the production of pro-inflammatory proteins via MAPK and the NF-κB pathway (Kawai and Akira, 2010). The MyD88 protein was discovered in 1990 (Lord et al., 1990) and since then has been widely studied. MyD88, together with TLR7 or TLR9, has been associated as a protective factor for immunity to malaria (Gowda et al., 2012; Spaulding et al., 2016). Gowda et al. showed that MyD88^-/-^ mice lacked cell-mediated immunity to malaria due to a reduction of pro-inflammatory proteins production. Additionally, the study shows that the MyD88 and TLR9 deficiency impaired NK and CD8^+^ T cell cytotoxic activity. Furthermore, other report showed that NK cell production of IFN-γ in response to *P. falciparum* infection is dependent of MyD88, which its expression in macrophages is crucial for an efficient response (Baratin et al., 2005). Accordingly, we have described that MyD88 and TLR4 signaling are fundamental to the placental inflammatory process induced by *Plasmodium* infection (Barboza et al., 2014, 2017).

Albeit the studies which agree that local inflammation in response to *Plasmodium* infection is crucial for the development of PM, little is known about the differential influence of maternal and fetal tissue to local immune response. Recently, Rodrigues-Duarte et al. (2018) presented results showing that fetal-derived TLR4 contributes to the iRBCs uptake by trophoblasts and to placental innate immune responses triggered in response to *P. berghei* infection.

Herein, we used an experimental mouse model that manifests several clinical features of the human disease in order to determine the contribution of maternal and fetal innate immune activation on PM pathogenesis. Therefore, we conducted a series of cross-breeding between mice with different MyD88 genotypes to generate several fetal genotypes born from mothers with distinct MyD88 backgrounds. Our findings have shown that the expression of fetal-derived MyD88 is detrimental for illness-induced complications of PM. These results extend and complete our previous observations showing the crucial role of MyD88 for local inflammation and consequent disease onset (Barboza et al., 2014).

## Materials and Methods

### Animals

C57Bl/6 WT, MyD88^+/-^, and MyD88^-/-^ mice with 8- to 10-weeks were bred and maintained in conventional housing with constant light-dark cycle (12 h:12 h) at the Animal Facility of the Department of Parasitology from the Institute of Biomedical Sciences at the University of São Paulo (ICB/USP). Mice received water and were fed *ad libitum* with commercial NUVILAB CR-1 ration (Nuvital^®^, Brazil). All experiments were performed in accordance with the ethical guidelines for experiments with mice, and the protocols were approved by the Animal Health Committee of the Institute of Biomedical Sciences of the University of São Paulo (CEUA No. 015fls82livro2). The guidelines for animal use and care were based on the standards established by the National Council for Control of Animal Experimentation (CONCEA).

### Cross-Breeding Experiments

**FIGURE 1:**
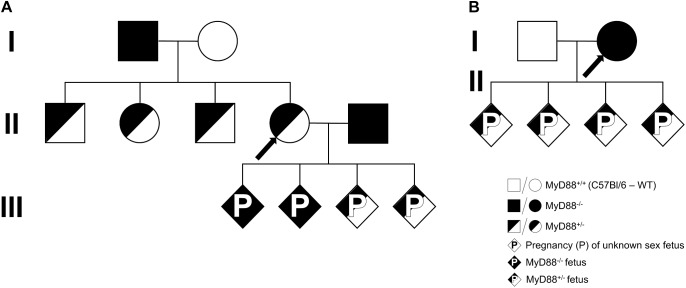
Breeding scheme. A schematic of the breeding strategy used to generate mice containing combinations of the maternal and fetal MyD88 genotypes. **(A)** MyD88 deficient (MyD88^-/-^) males (black square) mated with C57Bl/6 (MyD88^+/+^) females (white circle), generation **I**, to produce a heterozygous progeny (MyD88^+/-^, half-shaded symbols). MyD88^+/-^ females (half-shaded circle) from generation **II** were backcrossed with MyD88^-/-^ males (black square) to obtain fetus with MyD88^+/-^ (half-shaded diamond) and MyD88^-/-^ (black diamond) genotypes, generation **III**. **(B)** C57Bl/6 (MyD88^+/+^) males (white square) mated with MyD88 deficient (MyD88^-/-^) females (black circle), generation **I**, to obtain fetus with MyD88^+/-^ (half-shaded diamond) genetic background, generation **II**. Diamond represents pregnancy (P) of a fetus with unknown sex. The arrows depict the MyD88 maternal genotype for each MyD88 fetal genotype (**IIIA, IIB**).

To study the contribution of maternal and fetal MyD88 expression to PM development, heterogenic litters from females with heterozygous (Figure 1A) or null (Figure 1B) MyD88 expression were generated and then compared to WT and MyD88 full knockout fetuses/mothers. The generation of knockout (MyD88^-/-^) and heterozygous (MyD88^+/-^) fetuses from heterozygous mothers started by mating MyD88^-/-^ males with C57Bl/6 WT females (I from Figure 1A) to obtain a heterozygous progeny (II from Figure 1A). From these offspring, MyD88^+/-^ females were backcrossed with MyD88^-/-^ males (II from Figure 1A) and the fetuses and placentas (diamonds, sex not defined) from generation III were evaluated. Additionally, to obtain MyD88^+/-^ fetuses from MyD88^-/-^ mothers, we crossed C57Bl/6 WT males with MyD88^-/-^ females (I from Figure 1B), and the fetuses and placentas (diamonds, sex not defined) from generation II were evaluated.

### MyD88 Genotyping

To determine the genetic background of the generated offspring, tail genomic DNA was isolated, and genotyping was performed through conventional PCR using the following set of primers: Forward – 5^′^ TGG CAT GCC TCC ATC ATA GTT AAC C 3^′^; Reverse – 5^′^ GTC AGA AAC AAC CAC CAC CAT GC 3^′^; and, MyD88 neo – 5^′^ ATC GCC TTC TAT CGC CTT CTT GAC G 3^′^ (Leadbetter et al., 2002). The forward primer is common for both PCR reactions, while the reverse is specific for the wild-type gene and the neo is specific for the deficient gene. The PCR products were subjected to 1% agarose gel electrophoresis to verify the presence of a MyD88 allele (Supplementary Figure 1).

### Pregnancy Monitoring and Experimental Infection

Pregnancy was monitored as described elsewhere (Freyre et al., 2006). Briefly, we combine the detection of a vaginal plug and body weight measurement to determine the gestation time. The day in which a vaginal plug was detected was considered gestational day 1 (G1) and, from there on, pregnancy progression was monitored every day by regularly controlling weight variation. Successful fecundation was confirmed between G10 and G13 when females exhibited an average increase of 3–4 g in their body weight. Therefore, weight gain was taken as an indication of pregnancy, whereas unexpected weight loss was interpreted as an indicator of pregnancy complications or interruption. As such, pregnant mice were intravenously inoculated at G13 with 10^5^ *P. berghei* NK65^GFP^ iRBCs stored in frozen vials, and parasitemia was assessed. G13 was determined as the optimal time point for the infection, allowing to analyze the pathological features of malaria along the course of pregnancy and in the developing fetus. The infection at an earlier stage would not allow reaching the pregnancy term, which is consistent with previous reports (Neres et al., 2008; Marinho et al., 2009). Parasitemia was assessed by Giemsa-stained thin blood smears until G19, gestational day that cesarean section was performed. Fetal weight was measured, and placentas were used for histopathological analysis and RNA extraction. Non-pregnant infected and non-infected pregnant mice were used as controls whenever appropriate.

### Placenta Collection and Morphometric Analysis

Placentas from infected and non-infected pregnant mice were processed similarly. Briefly, placentas were divided into halves: one-half was fixed in 1.6% paraformaldehyde supplemented with 20% sucrose for further processing, and the other half was collected into an RNA stabilizer (RNAlater^TM^, Invitrogen^TM^, CA) for subsequent RNA extraction. Non-consecutive paraffin-embedded placentas were sectioned and stained with hematoxylin-eosin (H&E) for further microscope examination.

The placental morphometric analysis was performed as previously described (Marinho et al., 2009; Rodrigues-Duarte et al., 2012). In short, vascular spaces were quantified by analyzing the hematoxylin-eosin (H&E) stained sections. For each section, three areas of intervillous space were randomly selected for image acquisition (200× magnification) using a Zeiss color camera (Axio Cam HRc) connected to a Zeiss light microscope (Axio Imager M2). Images were analyzed using an Image J software^1^. Briefly, images were subjected to an automated light analysis procedure in which noise removal was applied to ensure color and image quality standardization across sections and specimens. Images were given a color threshold to cover the area corresponding to blood space lumen. Percentage of coverage was calculated as the ratio between the number of pixels covered by the threshold-defined area and the total amount of pixels in the image. Blood vascular area in each placenta was assessed from the analysis of three non-consecutive sections. Reported results correspond to individual pregnant mice, representing the average result from 3–9 placentas.

### Gene Expression Analysis by qPCR

Total RNA was extracted from each placenta obtained at G19 using the RNeasy Minikit (Qiagen) in accordance with manufacturer’s protocol for animal tissue (Animal Cell 1). One microgram of mRNA was converted into cDNA using First Strand cDNA Synthesis Transcriptor kit (Roche, Penzberg, Germany). The expression of *Il6*, *Il10*, *Tnf*, *Cxcl1* (KC), *Ccl3* (MIP-1α) and *Ccl4* (MIP-1β) was quantified by using the following TaqMan^®^ probes: *Il6* (Mm00446190_m1), *Il10* (Mm01288386_m1), *Tnf* (Mm00443258_m1), *Cxcl1* (Mm04207460_m1), *Ccl3* (Mm00441259_g1) and *Ccl4* (Mm00443111_m1). Gene expression quantifications were performed according to the manufacturer’s instructions on Applied Biosystems 7500 Fast Real-Time PCR System. All results were obtained through the comparative ΔΔCT method after normalization to the constitutive expression of the GAPDH gene (*gpdh*: Mm99999915_g1).

### Statistical Analysis

Statistical analysis was performed using the GraphPad Prism version 7.0 software (GraphPad Software, San Diego, CA, United States). To evaluate survival curves, the Long-rank test was used, the differences between groups were evaluated using the One or Two-way analysis of variance (ANOVA) as indicated at the figure’s legends. *P*-values < 0.05 were considered statistically significant.

## Results

### MyD88 Expression Influences the Progression of Murine Malaria Infection

**FIGURE 2:**
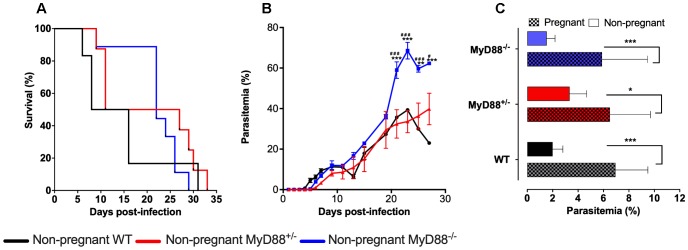
MyD88 expression influences disease progression in mice infected with *P. berghei* NK65. **(A,B)** Non-pregnant C57Bl/6 (WT), MyD88 deficient (MyD88^-/-^) and heterozygous (MyD88^-/-^) mice were infected intravenously with 10^5^ *P. berghei* NK65^GFP^ iRBCs. Evaluation of Kaplan-Meier survival curves **(A)** and parasitemia levels **(B)**. **(C)** Pregnant WT, MyD88^+/-^ and MyD88^-/-^ mice were intravenously infected at gestational day 13 (G13) with 10^5^ *P. berghei* NK65^GFP^ iRBCs. Parasitemia was measured at G19 before C-section and compared with non-pregnant mice, also with 6 days of infection. Plain bars represent non-pregnant mice, and square-patterned bars represent pregnant mice. In B and C data are presented as mean ± SD. The statistical differences were achieved by a Long-rank (Mantel-Cox) test **(A)** and Two-way analysis of variance (ANOVA) with the Bonferroni’s *post hoc* test **(B,C)**. ^∗∗^*P*-value <0.01 and ^∗∗∗^*P*-value < 0.001 when compared with WT **(B)** or non-pregnant mice **(C)**; ^#^*P*-value < 0.05 and ^###^*P*-value < 0.001 when compared with MyD88^+/-^ mice **(B)**.

In a previous study, we have shown that the MyD88 pathway is essential for the development of PM (Barboza et al., 2014). However, the results did not allow for individualizing the fetal- and maternal-derived MyD88 specific role in PM pathogenesis. To ascertain this, a series of experiments were conducted using C57Bl/6 (MyD88^+/+^ – WT), MyD88^+/-^ and MyD88^-/-^ mice, evaluating their survival and parasitemia progression upon infection with *P. berghei* NK65-iRBCs. The distinct groups of mice did not present significant differences regarding the survival rates when compared among each other (Figure 2A). However, it was possible to note that the MyD88^-/-^ group started to die later, probability due to the augment of parasitemia levels observed from the twentieth day post-infection onward (Figure 2B).

The evaluation of parasitemia did not show significant differences, with MyD88^+/-^ mice behaving similarly to the WT group (Figure 2B). Moreover, our data corroborate previous studies showing that pregnancy increases mice susceptibility to infection, irrespective of the maternal genotype (Figure 2C).

**FIGURE 3:**
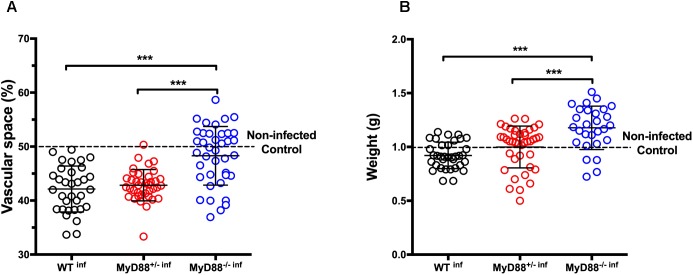
MyD88 expression is associated with reduced placental vascular space and fetal weight in mice infected with *P. berghei* NK65. Pregnant C57Bl/6 (WT), and MyD88 deficient (MyD88^-/-^) and heterozygous (MyD88^-/-^) mice were intravenously infected (^inf^) with 10^5^ *P. berghei* NK65^GFP^ iRBCs at gestational day 13 (G13) and C-section performed at G19. The placenta/fetus share the same maternal MyD88 genotype. **(A)** Placental vascular spaces (n: WT^inf^ – 33; MyD88^+/-inf^ – 38; MyD88^-/-inf^ – 39) and **(B)** fetal weight (n: WT^inf^ – 35; MyD88^+/-inf^ – 40; MyD88^-/-inf^ – 28) measures. Data are presented as scatter plot with indication of the median ± SD; each dot represents an independent measurement. Dotted line represents mean values of the control group (non-infected pregnant WT mice; n: 34, vascular space, and 32, fetal weight). The statistical differences were achieved by a One-way analysis of variance (ANOVA) with *post hoc* Tukey’s test. ^∗∗∗^*P*-value < 0.001.

Although there were no differences between peripheral parasitemia of pregnant mice with different genotypes, we hypothesized that heterozygous and null MyD88 expression might be involved in placental damage and poor pregnancy outcomes. Therefore, we evaluated the placental vascular spaces as an indicator for placental abnormalities. The expression of only one MyD88 allele is sufficient to induce placental structural changes as similarly to WT infected mice (WT^inf^), MyD88^+/-^ infected mice (MyD88^+/-inf^) presented reduced vascular spaces (Figure 3A). On the other hand, the absence of the two MyD88 alleles (MyD88^-/-inf^) showed no infection deleterious impact in the placental vasculature, identical to the non-infected controls (dashed line). Moreover, fetal weight exhibited a similar pattern. Like fetuses born from WT^inf^, the progeny from heterozygous MyD88 pregnant mice (MyD88^+/-inf^) also presented reduced fetal weight upon infection. Notably, in the fetuses born from MyD88^-/-^ infected mice malaria did not impair their weight when compared to WT^inf^ and MyD88^+/-inf^ mice (Figure 3B). The fetal weight in these fetuses reached values resembling those from healthy WT mice.

### MyD88 Expression on Fetal-Derived Tissue Is Detrimental for Pregnancy Outcomes

**FIGURE 4:**
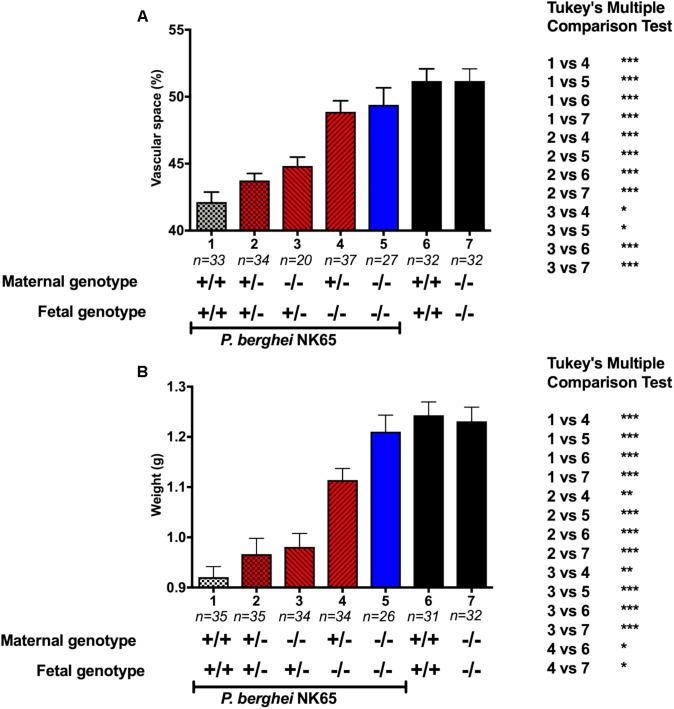
Fetal MyD88 expression is detrimental to disease development in an experimental mouse model of PM. To ascertain the differential contribution of the maternal- and fetal-derived MyD88 expression different combinations of maternal/fetal genotypes were evaluated. Pregnant mice were intravenously infected with 10^5^ *P. berghei* NK65^GFP^ iRBCs at gestational day (G13), with the following maternal-fetus pairs: WT/WT (1), MyD88^+/-^/MyD88^+/-^ (2), MyD88^-/-^/MyD88^+/-^ (3), MyD88^+/-^/MyD88^-/-^ (4), and MyD88^-/-^/MyD88^-/-^ (5). C-section was performed at G19 and fetus were genotyped and evaluated. **(A)** Placental vascular spaces and **(B)** fetal weight measures. Infected WT maternal-fetus pair is a positive control (1) and non-infected WT and MyD88^-/-^ maternal-fetus pairs are negative controls (6 and 7, respectively). Data are presented as mean ± sem. The statistical differences were achieved by a One-way analysis of variance (ANOVA) with Tukey’s *post hoc* test. ^∗^*P*-value < 0.05; ^∗∗^*P*-value < 0.01; ^∗∗∗^*P*-value < 0.001.

To investigate the role of maternal- and fetal-derived MyD88 in PM-associated pregnancy outcomes. We performed a comparative analysis between placental vascular spaces (Figure 4A) and fetal weight (Figure 4B) associated with different MyD88 genotypes, pairing maternal and fetal genetic backgrounds. Including an infected WT maternal-fetus pair as a positive control (group 1) and as negative controls a non-infected WT and MyD88^-/-^ maternal-fetus pairs (group 6 and 7, respectively). The results demonstrate that the presence of only one fetal-derived MyD88 allele is sufficient to contribute to a prominent reduction of placental vascular spaces (Figure 4A, groups 2 and 3) and fetal weight (Figure 4B, groups 2 and 3) upon *P. berghei* NK65 infection when comparing to full knockout fetuses (Figure 4, group 4 and 5). Also, these results show that there are no influence of maternal MyD88 genotype since MyD88^-/-^ fetuses from infected MyD88^+/-^ did not have a negative impact on either vascular spaces or fetal weight (Figure 4, group 4).

To validate our results, we performed a second multivariable statistical analysis, excluding the control groups (1, 6, and 7) from the analysis. The new analysis reinforced the idea that the fetuses genotype has a significant effect on both fetuses’ weight and the placental vascular space while the maternal genotype does not (Supplementary Figure 2 and Supplementary Table 1). Together, our results support the idea that fetal-derived MyD88 is associated with local parasite sensing, triggering inflammation and, consequently leading to poor pregnancy outcomes and PM development. Interestingly, the deletion of one maternal MyD88 allele was sufficient to negatively modulate the expression of placental inflammatory cytokines mRNA, except for the *Il10* (Supplementary Figure 3), indicating the possible involvement of other mechanisms.

## Discussion

Innate immune activation during pregnancy represents a substantial problem for the mother and the growing fetus. Regarding *Plasmodium* infection, it has been shown that innate immune activation in response to parasite accumulation results in placental injuries, which ultimately leads to impaired fetal development. Previously, we have demonstrated that MyD88 signaling is associated with poor pregnancy outcomes (Barboza et al., 2014, 2017). Other studies also have unveiled that the MyD88 protein plays an important role in inducing immune protection to *Plasmodium* infection (Baratin et al., 2005; Gowda et al., 2012; Spaulding et al., 2016). Herein, using mice cross-breeding strategies, we managed to obtain distinct MyD88 genetic backgrounds and determine the maternal- and fetal-derived MyD88 contribution to PM. Our results extend previous observations, showing that fetal-derived MyD88 is markedly associated with murine PM development.

Since the placenta is constituted by cells from at least two different individuals, one can argue that those cellular components can differentially influence placental physiology. The results presented here evidence the contribution of fetal-derived MyD88 expression in the disease onset, highlighting the influence of fetal components in detriment of the maternal components. Such impact is perceived when we evaluate placental vascular spaces of infected pregnant mice. Our work revealed that placental vascular spaces reduction upon infection is mediated by fetal-derived MyD88, even when only one allele is carried (Figure 4A group 2 and 3 and Supplementary Figure 2). Of note, the importance of this observation is supported by the minor impact of maternal MyD88 expression (Figure 4A group 4 and Supplementary Figure 2). Previously, it was described that in murine PM the reduction of placental vascular spaces is correlated with low birth weight (Neres et al., 2008; Marinho et al., 2009; Medeiros et al., 2013; Barboza et al., 2014). In fact, we also observed a parallel reduction of fetal weight, which was equally determined by fetal-derived MyD88 expression (Figure 4B group 2 and 3). We should emphasize that the deletion of the MyD88 gene does not impact the mice growth nor produces abnormalities (Adachi et al., 1998), as well as have no influence in pregnancy in general, as non-infected MyD88^-/-^ and MyD88^+/-^ have no differences in the pregnancy outcome.

In line with our observation, Rodrigues-Duarte et al. (2018) have recently reported that maternal and fetal counterparts act in opposite directions during murine PM. This study showed that TLR4 and IFNAR1 expression in the maternal tissue has a deleterious effect on fetal development, which is counteracted by fetal tissue. The role of these family of innate immune receptors on PM has been extensively studied in humans and murine malaria models (Adegnika et al., 2008; McDonald et al., 2015; Odorizzi and Feeney, 2016; Barboza et al., 2017; Rodrigues-Duarte et al., 2018). These studies have shown that local immune activation via innate immune receptors is correlated with placental damage, which results in impaired fetal development.

The negative impact of innate immune activation observed in the fetuses is correlated with a rupture of maternal-fetal interactions during pregnancy. For instance, to achieve a successful pregnancy it is necessary that trophoblasts (fetal-derived cells which populate most of the placenta) and maternal decidual immune cells interact, allowing the development of the embryo in the uterus (Prabhudas et al., 2015). One way of promoting this rupture is through the activation of MyD88-dependent pathways induced by TLR activation in trophoblasts, which are known to broadly express TLRs (Abrahams et al., 2004; Mitsunari et al., 2006; Lucchi and Moore, 2007; Aldo et al., 2010; Tangerås et al., 2014). Moreover, TLR activation has been associated with pregnancy complications (Mockenhaupt et al., 2006; Rindsjö et al., 2007; Leoratti et al., 2008; Hamann et al., 2010; Pineda et al., 2011; Koga et al., 2014). Thus, TLR activation by the parasite components can lead to NF-κB activation and, as a consequence, to the production of pro-inflammatory mediators such as chemokines and cytokines, contributing to tissue damage and PM development.

Nevertheless, our work may present some limitations. The PM murine models recapitulate many features observed in the human’s disease, which represent numerous advantages in the research and acquisition of knowledge of this disease (Hviid et al., 2010). Though, human and mouse pregnancy differences do not allow us to make direct assumptions, such as the placental architecture or the immunological responses (Moffett and Loke, 2006; Carter, 2007). Hence, it would be of crucial relevance the validation of murine results with human samples, whenever is possible. Following our results in murine models, it would be interesting to ascertain the correlation of the maternal and fetal genetic interplay with pregnancy outcomes upon *Plasmodium* infection.

Our results on the placental vasculature and fetal weight obtained using heterogenic mice progeny from different maternal MyD88 genetic backgrounds have evidenced a marked impact of the fetal immune system in the PM onset. In summary, this study highlights the importance of maternal and fetal immune response in the context of pregnancy-associated malaria, which ultimately contributes to the placental pathology and poor pregnancy outcomes.

## Author Contributions

RB and CM designed the study. LH, RB, OM, AB, EP, FL, AR, LG, SE, and CM were involved in data acquisition and scientific input. RB, LH, LG, SE, and CM contributed to the analysis and/or interpretation of the data. RB, LG, and CM wrote the manuscript. SE revised the manuscript. CM, SE, and RB were the main funders of this work. CM has full access to all the data in the study and takes responsibility for the integrity of the data and the accuracy of the data analysis. All authors reviewed and approved the definitive version of this manuscript.

## Conflict of Interest Statement

The authors declare that the research was conducted in the absence of any commercial or financial relationships that could be construed as a potential conflict of interest.

## Footnotes

1 http://rsbweb.nih.gov/ij/
