# Supplementary material for: Fetal-Derived MyD88 Signaling Contributes to Poor Pregnancy Outcomes During Gestational Malaria
Source: Front Microbiol. 2019 Jan 29;10:68. doi: 10.3389/fmicb.2019.00068 (PMC6362412; doi:10.3389/fmicb.2019.00068)
Supplement: Supplementary file 1 [file Data_Sheet_1.PDF]

Supplementary Material

Fetal-derived MyD88 signaling contributes to poor pregnancy outcomes during gestational malaria

Renato Barboza, Lutero Hasenkamp, André Barateiro, Oscar Murillo, Erika Paula Machado Peixoto, Flavia Afonso Lima, Aramys Silva Reis, Lígia Antunes Gonçalves, Sabrina Epiphanio and Claudio Romero Farias Marinho*

*** Correspondence:** Cláudio RF Marinho, marinho@usp.br

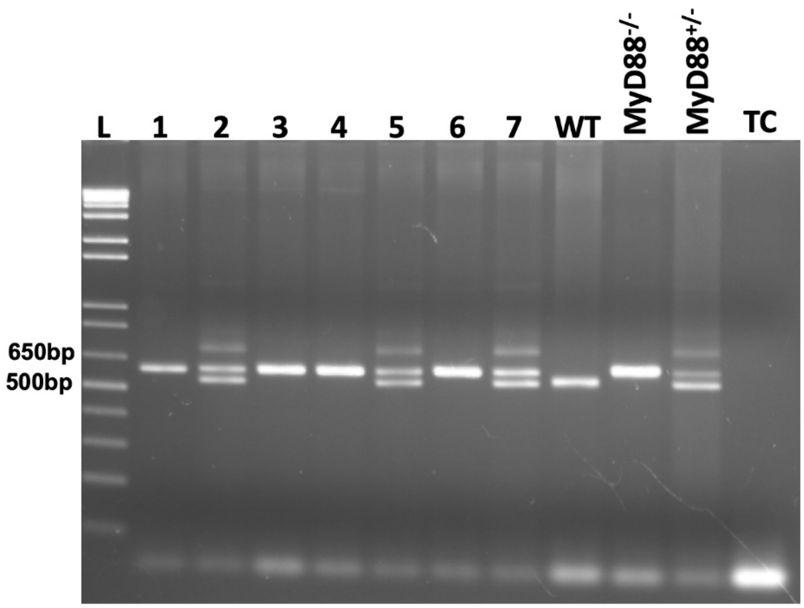

Supplementary Figure 1. Representative image of gel electrophoresis of the mice MyD88 genotype screening. Wildtype (WT) mice were identified by a single 500pb band, MyD88 deficient mice (MyD88$^{-/-}$) by the presence of a single 550-bp and heterozygous mice (MyD88$^{+/-}$) by the presence of both bands. Line 1 to 7 represent samples. **L**: 1Kb DNA Ladder; **TC**: Template control.

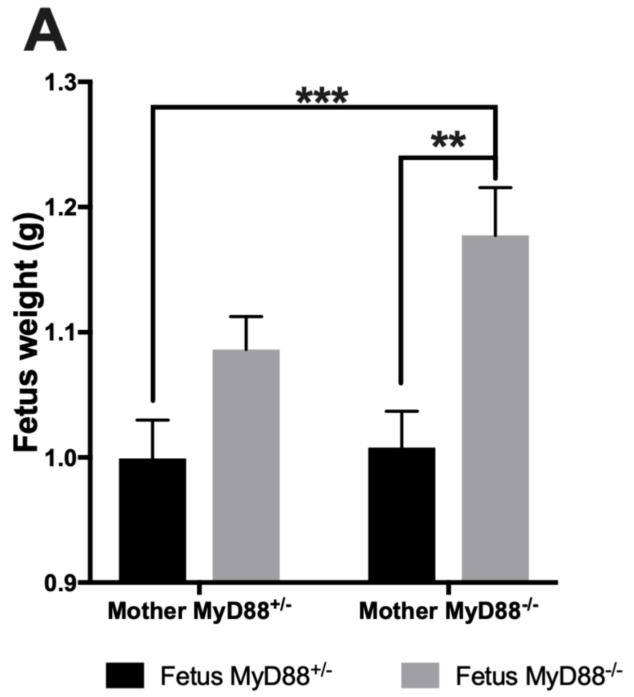

Supplementary Figure 2. Fetal-derived MyD88 contributes to the detrimental effects observed in the experimental mouse model of PM. Analysis of fetus and mother genotype effect on fetuses' weight (**A**) and vascular space (**B**). Data are presented as mean±sem. The statistical differences were achieved by a Two-way analysis of variance (ANOVA) with Tukey´s multiple comparisons test. ** P-value < 0.01; *** P-value < 0.001.

Supplementary Table 1. Two-way ANOVA with genotype as main factors.

	Source of Variation	Sum of Square	DF	F	P value
Fetus weight					
	Fetus	0.5737	1	17.14	*P<0.0001*
	Mother	0.08713	1	2.603	*P=0.1089*
	Interaction	0.05927	1	1.771	*P=0.1855*
Vascular space					
	Fetus	571.1	1	19.8	*P<0.0001*
	Mother	15.42	1	0.5906	*P=0.4437*
	Interaction	4.384	1	0.1678	*P=0.6827*

Statistical table is showing two-way ANOVA results for analyses of fetus and mother genotype effect on fetuses' weight and vascular space.

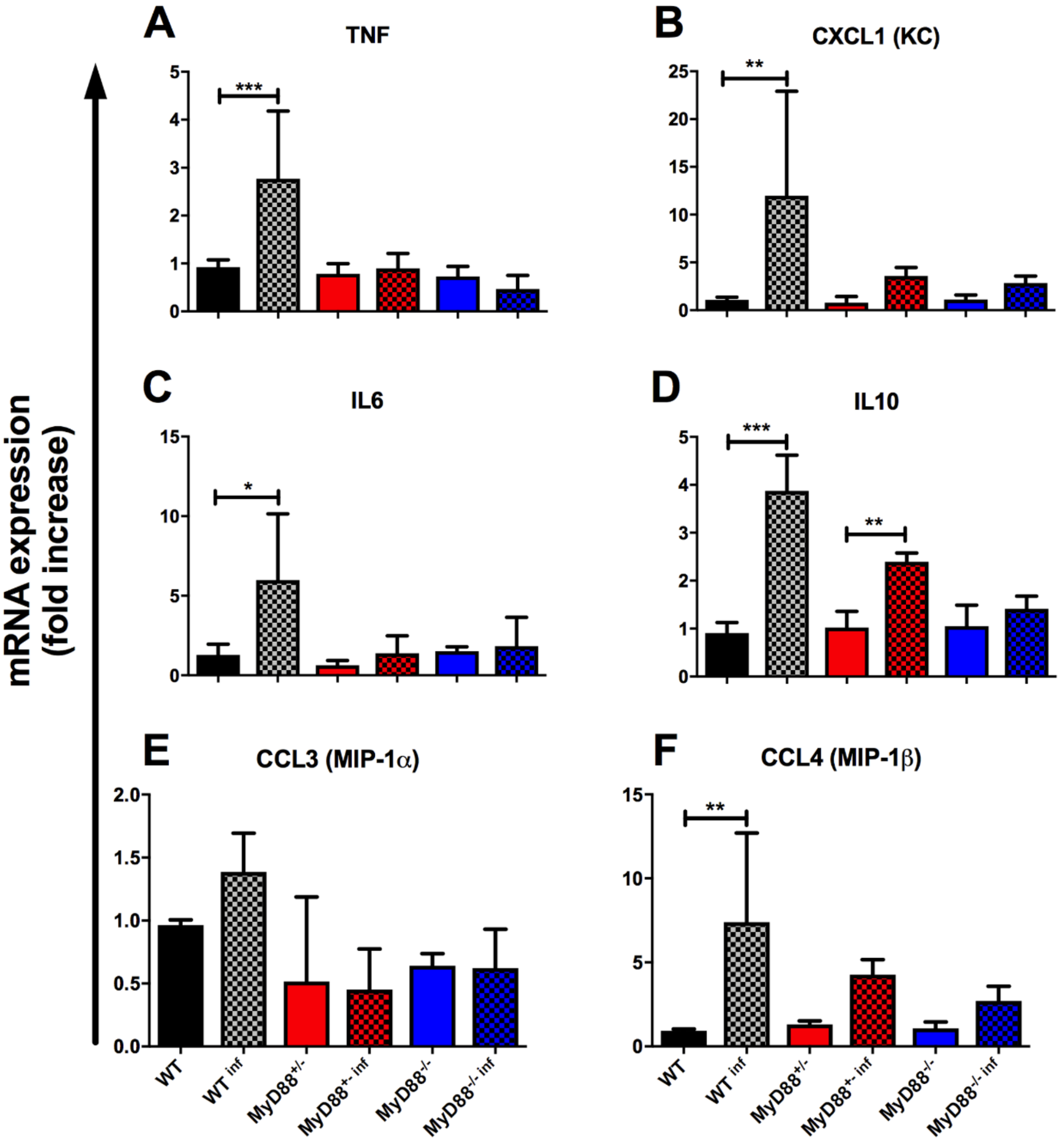

Supplementary Figure 3. Placental inflammatory response is driven by homozygous MyD88 expression in *P. berghei* infection. mRNA expression of TNF-α **(A)**, CXCL1 (KC) **(B)**, IL-6 **(C)**, IL-10 **(D)**, CCL3 (MIP-1α) **(E)** and CCL4 (MIP-1β) **(F)** was quantified in placentas collected from pregnant C57Bl/6 (WT), and MyD88 deficient (MyD88$^{-/-}$) and heterozygous (MyD88$^{+/-}$) mice intravenously infected (^{inf}) with 10^5 *P. berghei* NK65^{GFP} iRBCs at gestational day 13 (G13) and C-section performed at G19. The placenta shares the same maternal MyD88 genotype. Plain bars represent the non-infected mice and bars, and square-patterned bars represent the infected mice. Results were plotted as fold-change to non-infected pregnant WT mice; each bar represents the mean ± SD (5 placentas/group from different donors). The statistical differences were achieved by a Two-way analysis of variance (ANOVA) with Bonferroni's post hoc test. * P-value < 0.05; ** P-value < 0.01; *** P-value < 0.001.

Material and Methods:

Total RNA was extracted from each placenta obtained at G19 using the RNeasy Minikit (Qiagen) in accordance with manufacturer's protocol for animal tissue (Animal Cell 1). One microgram of mRNA was converted into cDNA using First Strand cDNA Synthesis Transcriptor kit (Roche, Penzberg, Germany). The expression of *Il6*, *Il10*, *Tnf*, *Cxcl1* (KC), *Ccl3* (MIP-1α) and *Ccl4* (MIP-1β) was quantified by using the following TaqMan® probes: *Il6* (Mm00446190_m1), *Il10* (Mm01288386_m1), *Tnf* (Mm00443258_m1), *Cxcl1* (Mm04207460_m1), *Ccl3* (Mm00441259_g1) and *Ccl4* (Mm00443111_m1). Gene expression quantifications were performed according to the manufacturer's instructions on Applied Biosystems 7500 Fast Real-Time PCR System. All results were obtained through the comparative ΔΔCT method after normalization to the constitutive expression of the GAPDH gene (*gpdh*: Mm99999915_g1).

frontiers
